# The History of Medical Education, Etc.

**Published:** 1851-05

**Authors:** 


### THE WESTERN JOURNAL.

THIRD SERIES.—VOL. VII.—NO. V.

LOUISVILLE, MAY 1, 1851.

### THE HISTORY OF MEDICAL EDUCATION, ETC.

Dr. Davis, of Chicago, has considered it necessary to respond to the notices of his work, on medical education, which appeared in the New York Journal of Medicine, and in this *Journal.* We did not undertake to review the book of Dr. Davis, but was content with an expression of the judgment we gave, founded on a perusal of the work. Dr. Davis is not pleased with the character of that judgment, but we assure him it was honestly formed, and we do not now find, after reading the rejoinder of Dr. Davis, any reason to retract or modify the opinion we formerly expressed.

We alluded to the representation made by Dr. Davis of the dearth of medical literature, in this country, at a specified period of American history. The New York Journal published a list of American books, which were printed during the blank referred to by Dr. Davis, but Dr. Davis says he had actually quoted from some of these very books. These quotations made the following declaration the more singular: "the medical writings of this period seem to have been confined almost wholly to the pages of the three medical journals which had been established in New York and Philadelphia, and to here and there a pamphlet, or a paper read before some organized society. Indeed, I have not been able to find a single *volume* on any *branch* of medical science or practice, published by an American physician during the first twenty years after the close of the revolutionary war. And yet, as we have already seen, it was a period in our history which was graced by some of *the noblest and most active minds ever devoted to the cultivation of medical science.* But the art of *mere book making*, which has been brought to such perfectiou in this our day, was little known to our professional ancestors."

Dr. Davis thinks his meaning should have been "obvious to the most careless reader," and we cheerfully admit that just that kind of a reader might easily have stumbled into the meaning Dr. Davis now gives his language. But we do not belong to the class of readers, upon which Dr. Davis leans for countenance in this matter. We read the language carefully, and we could not then, nor can we now, put any other construction on it than that we have given. We are bound to believe that "Collections for an essay towards a Materia Medica of the United States, by Dr. Barton, commenced in 1798," "Observattions on the causes and cure of remitting bilious fevers," by Dr. Currie," "A treatise on yellow fever, by Dr. Davidge, of Baltimore," "Letters on yellow fever, by Richard Bagley, New York," all published in 1798, and a work "On the origin of pestilential diseases, by Dr. Charles Caldwell," published in 1799, to say nothing of other books, were volumes on some branches of medical science and practice. Dr. Davis seems to think that because he said that the period embraced in his non-book-making time, "was graced with some of the noblest and most active minds ever devoted to the cultivation of medical science," he should have been understood to mean that these active minds were given to book making, when he immediately adds that they were not, and when he prefaces his compliment with the declaration that, he had "not been able to find a single *volume* on any *branch* of medical science or practice, published by an American physician during the first twenty years after the close of the revolutionary war." And Dr. Davis would have careful, as well as "careless readers" to understand him as meaning, that the books referred to had nothing to do with "physiology, midwifery, materia medica, practical medicine, etc." This may do in regions where the mysteries of a fox chase are better understood practically, than we can be supposed to understand them, and we assure Dr. Davis that we are utterly unable to perceive, in any degree whatever, how his explanation relieves him. If he thinks it helps his cause, we are yet even with him, for we think it does not.

The History of American Medical Education is a grand theme, filled with great results, and some of the brightest triumphs of science, and we should rejoice to see justice done to the noble subject.

Since the preceding remarks were written, we have received the May number of the *Northern Lancet*, containing a series of very impertinent and unjust remarks upon the American medical press. We think that we have enjoyed a good opportunity of knowing the character of the conductors of the medical press in this country, and from our long and tolerably intimate acquaintance with an extensive range of the "humanities" of the times, we declare that we know of no class of persons more thoroughly honest, intelligent, and worthy, than the great mass of the editors of the American Medical Journals. It is seldom that asperities and personalities are indulged in, and we believe there are few medical editors who permit their judgment to be swayed by anything but honest convictions. The utterly disgraceful "caricature and libel" upon the editorial corps, contained in the defence, set forth in the *Northern Lancet*, of Dr. Davis's *History*, needs some apology, since it is without any provocation.

In another part of the model for medical reviewing, contained in the defence referred to, the urbane, polite, and genteel editor of the *Northern Lancet* thus holds forth : "The work (Dr. Davis's *History*) was greedily seized upon by a few of our contemporaries, who, in their criticisms, evidently dictated by animosity and personal feeling, misconstrued the author's intentions," [another Daniel come to judgment!] "and from glancing over one or two pages, inferred the whole, and as it should be expected, the notice turned out to be in perfect accordance with the spirit that presided over it."

After having uttered this string of gratuitous calumnies upon those who noticed *the* History, our amiable confrere of the *Lancet* lustily boasts that he has honesty enough to admire Dr. Davis's book. What a pity he had not some honesty to spare for other purposes!

We thought we dealt mercifully with Dr. Davis's book, and it may be yet found that we did, if we are compelled to prove it. B.

